# Correspondence between the Simple Physical Activity Questionnaire (SIMPAQ) and accelerometer-based physical activity in inpatients treated for major depressive disorders in comparison to non-depressed controls

**DOI:** 10.3389/fspor.2024.1447821

**Published:** 2024-09-06

**Authors:** René Schilling, Robyn Cody, Jan-Niklas Kreppke, Oliver Faude, Johannes Beck, Serge Brand, Lars Donath, Martin Hatzinger, Christian Imboden, Undine Lang, Sarah Mans, Thorsten Mikoteit, Anja Oswald, Nina Schweinfurth-Keck, Markus Gerber

**Affiliations:** ^1^ Department of Sport, Exercise and Health, University of Basel, Basel, Switzerland; ^2^ Psychiatric Clinic Sonnenhalde, Riehen, Switzerland; ^3^ Center for Affective, Stress and Sleep Disorders (ZASS), Psychiatric University Hospital of the University of Basel, Basel, Switzerland; ^4^ Sleep Disorders Research Center, Kermanshah University of Medical Sciences (KUMS), Kermanshah, Iran; ^5^ Substance Abuse Prevention Research Center, Kermanshah University of Medical Sciences (KUMS), Kermanshah, Iran; ^6^ School of Medicine, Tehran University of Medical Sciences, Tehran, Iran; ^7^ Department of Training Intervention Research, German Sport University Cologne, Cologne, Germany; ^8^ Psychiatric Services Solothurn and Faculty of Medicine, University of Basel, Solothurn, Switzerland; ^9^ Private Clinic Wyss, Münchenbuchsee, Switzerland; ^10^ Translational Research Center, University Hospital of Psychiatry and Psychotherapy, University of Bern, Bern, Switzerland; ^11^ Adult Psychiatric Clinics (UPKE), University of Basel, Basel, Switzerland

**Keywords:** assessment, exercise, measurement, mental illness, physical activity, sedentary behaviour, accelerometry, fitness

## Abstract

**Introduction:**

Major depressive disorders (MDD) are a leading health concern worldwide. While first line medication treatments may fall short of desired therapeutic outcomes, physical activity (PA) interventions appear to be a promising and cost-effective add-on to improve symptoms of depression. This study aimed to address challenges in the assessment of PA in inpatients treated for MDD by examining the correspondence of self-reported and accelerometer-based PA.

**Methods:**

In 178 inpatients treated for MDD (mean age: *M* = 41.11 years, SD = 12.84; 45.5% female) and 97 non-depressed controls (mean age: *M* = 35.24 years, SD = 13.40; 36.1% female), we assessed self-reported PA via the Simple Physical Activity Questionnaire (SIMPAQ) for one week, followed by a week where PA was monitored using an accelerometer device (Actigraph wGT3x-BT). Additionally, we examined correlations between PA levels assessed with the SIMPAQ and exercise determinants in both groups.

**Results:**

Descriptively, inpatients treated for MDD showed lower levels of light PA on accelerometer-based measures, whereas they self-reported increased levels of certain types of PA on the SIMPAQ. More importantly, there was only a small degree of correspondence between self-reported and actigraphy-based PA levels in both in patients (*r* = 0.15, *p* < 0.05) and controls (*r* = 0.03, ns). Only few significant correlations were found for self-reported PA (SIMPAQ subscores) and perceived fitness, whereas self-reported PA and estimated VO_2_max were unrelated. Furthermore, only weak (and mostly statistically non-significant) correlations were found between exercise determinants and SIMPAQ-based exercise behavior in both populations.

**Discussion:**

Our findings emphasize the intricate challenges in the assessment of PA, not only in inpatients treated for MDD, but also in non-depressed controls. Our findings also underline the necessity for a diversified data assessment. Further efforts are needed to refine and improve PA questionnaires for a more accurate data assessment in psychiatric patients and healthy controls.

## 1 Introduction

Major depressive disorders (MDD) are a leading health concern, ranking second in terms of years lost due to disability or premature death (DALY), and topping the list for years lived with disability for both genders, as outlined by the World Health Organization (1). As the prevalence of MDD escalates (2), the financial implications are felt profoundly, with tremendous treatment costs (3). The increasing burden of MDD underscores an urgent need for effective interventions.

Physical activity (PA) has been substantiated as a cost-effective and impactful intervention for ameliorating symptoms of depression, with effects comparable to that of antidepressants and psychotherapy (4, 5). Meta-analytical data reinforce these findings, with evidence of moderate to large significant effects of exercise training on reducing depressive symptoms compared to control conditions (6). Specific studies have shown that aerobic exercise for as little as 30 min a day over 10 days can produce substantial improvements in mood in patients with MDD (7).

PA plays a crucial role in mitigating the morbidity and mortality associated with MDD. Depressive symptoms have been associated with an increased mortality risk, a relationship partially explained by factors such as physical inactivity, physical illnesses, and impairments in physical and cognitive functioning (8). In older adults with depressive symptoms, physical inactivity significantly elevates the risk of cardiovascular mortality, regardless of coronary heart disease status (9).

Furthermore, patients with MDD exhibit altered autonomic function during resting conditions, exercise, and recovery from exercise, which may influence their cardiovascular morbidity and mortality (10). PA, particularly (regular) aerobic exercise, such as walking or cycling, performed at moderate intensity for at least 30 min per session, three to five times a week, can enhance the neuroprotective effect of antidepressants, potentially preventing cardiovascular diseases in patients with MDD (11). Exercise-based interventions have also been effective in reducing cardiovascular risk and mortality in depressed populations, while simultaneously improving depressive symptoms (12).

Studies indicate that PA, both as a stand-alone and as an add-on to standard treatment, is effective in reducing depressive symptoms. For instance, Blumenthal and colleagues (13, 14) showed significant improvements in depressive symptoms with exercise intervention. Dunn et al. (15) also found that exercise has a dose-response effect on depression symptoms.

Additionally, PA as an add-on to standard treatment has been shown to enhance treatment outcomes. Schuch et al. (16) reported preliminary results indicating that exercise can be an effective add-on treatment for severe depression. Imboden and colleagues (17) found similar antidepressant effects for aerobic exercise and stretching when added to inpatient treatment, with larger effects on working memory for aerobic exercise alone. Furthermore, Ravindran and da Silva (18) highlighted the benefits of complementary and alternative therapies, including exercise, as add-ons to pharmacotherapy for mood and anxiety disorders.

In sum, PA, including exercise and sport, emerges as a cost-effective and impactful add-on intervention to improve symptoms of depression beyond standard treatment (19, 20). Research indicates that combining PA with standard treatment results in a greater effect size compared to either treatment alone, making this combined approach more effective in reducing depressive symptoms and improving overall health outcomes (21).

While there are many tools available to assess PA (22), challenges remain in their application to specific populations such as those with MDD. In light of this, the importance of accurate assessment tools for PA within the MDD patient population has been emphasized (20). The current lack of stratification tools for the risk of physical inactivity is believed to pose a significant obstacle for clinicians to assess PA and plan specific interventions and coaching to improve PA and exercise participation (23). For clinicians, it is important to have access to brief and precise measures to monitor health factors over time, particularly as patients tend to be more compliant (24), report less missing data or errors due to fatigue or frustration (25) when assessment tools are less time-consuming.

PA is most commonly assessed via accelerometers and questionnaires (26, 27). While accelerometers provide objective data on PA behavior, they are unable to assess the context of PA and certain movements such as rowing, weight lifting, and bike riding. There is also a risk of measurement error due to non-wear time. Additionally, accelerometers are costlier than questionnaires, and they pose a higher burden for patients (wear time between days and weeks; personal privacy) as well as for clinicians, who need time and training to analyze the data. Accordingly, accelerometry is still not used very often in clinical practice (28–30). Questionnaires, on the other hand, are easy to use and bear relatively low costs (31). However, they rely on the respondent's ability to accurately recall the behavior of interest, a capability which may be impaired in patients with MDD (32). There is also a risk that data are biased due to social desirability. The poor agreement between the IPAQ (International Physical Activity Questionnaire) and ActiGraph data, as previously reported by do Nascimento et al. (33), highlights a significant gap in current assessment methodologies. It should also be noted that no distinction has been made in previous meta-analyses between device-based and self-reported PA when looking at the potential of PA to reduce the risk of mental illnesses, thus assuming that there is a sufficient degree of correspondence between both assessment techniques (34, 35).

Given this background, the aim of this paper is to address this research gap by examining the correspondence between self-reported and device-based PA measures in patients with MDD. More specifically, this paper will (i) examine in inpatients treated for MDD and non-depressed controls whether and to what degree self-reported PA (assessed via SIMPAQ) would be associated with accelerometer-based PA, (ii) explore how self-reported PA correlates with perceived fitness and estimated VO_2_max, and (iii) examine the relationships between SIMPAQ-based exercise behavior and selected cognitive exercise determinants including exercise-based self-efficacy, outcome expectancies, exercise intention, and intrinsic exercise motivation. The present study adds to the current literature in so far that few studies have provided data regarding the correspondence of the SIMPAQ with objective measures of PA. It should also be noted that most PA questionnaires have been tested in healthy and active populations (27), whereas in the present study, we focused on inpatients treated for MDD and initially insufficiently physically active individuals.

In a previous study with college students, we demonstrated a moderate-to-high correlation between the SIMPAQ and accelerometer data (*r* = 0.30–0.70) (36). In a separate study involving 1,010 participants with mental illness, the SIMPAQ exhibited good test-retest reliability (*r* = 0.63–0.76). Regarding MVPA, the overall correlation with accelerometer-based data was *r* = 0.25, with the correlation being lower for inpatients (*r* = 0.09) compared to outpatients (*r* = 0.43) (23). In another study, the SIMPAQ demonstrated sensitivity in detecting changes resulting from a PA counselling intervention (37).

As stated above, the ability to recall behavior accurately may be impaired to some degree in inpatients with MDD (32). Regarding our hypotheses, we took this finding into consideration, as well as previous evidence based on participants with MDD (33), and healthy samples, in which the SIMPAQ has been applied (36). As a consequence, we formulated the following cautious hypothesis:

We expected weak to moderate correlations between self-reported (SIMPAQ-based) and accelerometer-based PA in both inpatients treated for MDD and non-depressed controls (Hypothesis 1) (23, 32, 33). We further hypothesized that self-reported PA would be positively associated with perceived fitness and estimated VO_2_max (Hypothesis 2) (38). Finally, we assumed that the SIMPAQ (particularly the exercise/sport subscore) would be moderately correlated with cognitive exercise determinants such as self-efficacy, positive outcome expectancies, exercise intention, and intrinsic exercise motivation (Hypothesis 3) (39, 40).

## 2 Methods

### 2.1 Design

The present paper was based on baseline data from a multi-centric, two-arm randomized controlled trial (RCT), including an intervention group (extended personalized PA and exercise counselling program), a placebo control group (general instructions about health-enhancing PA) (19), and a non-depressed control group (being invited to the baseline assessments only). The study was initiated by the Department of Sport, Exercise and Health of the University of Basel, and carried out in cooperation with four Swiss psychiatric clinics (2 public, 2 private).

### 2.2 Participants

Recruitment lasted from June 2019 to October 2021. Based on a structured clinical interview conducted by two trained researchers, all inpatients fulfilled ICD-10 diagnosis for single episode (F32), recurrent MDD (F33) or bipolar disorder type II (F31.80). As part of the screening process, the 17-item Hamilton Depression Rating Scale (HAMD17) (41) was applied by a trained staff member, whereas inpatients completed the 21-item Beck Depression Inventory-II (BDI-II) (42, 43) to assess symptom severity. Information on PA during the last week before entering the clinic was collected via the short version of the International Physical Activity Questionnaire (IPAQ) (44). To be eligible for the present study, inpatients had to meet the following inclusion criteria: (a) inpatients treated for depression on a specialized ward at one of the study centers, (b) 18–65 years of age, (c) presence of MDD according to ICD-10 diagnostic criteria, (d) BDI ≥17 (at least self-rated moderate MDD), (e) current PA level assessed via IPAQ of less than 150 min/week of moderate-to-vigorous PA (MVPA), (f) signed written informed consent, and (g) ability to speak and read German and to comply with the study conditions. A sample of non-depressed controls was recruited via advertisements in newspapers and word-of-mouth recommendation. For non-depressed controls, the following inclusion criteria were considered: (a) 18–65 years of age, (c) HAMD17 ≤7, (d) BDI ≤13, (e) current MVPA level of less than 150 min/week (IPAQ), (f) written informed consent, and (g) ability to speak and read German.

In total, 210 inpatients and 125 non-depressed controls met all inclusion criteria and provided SIMPAQ and accelerometer-based data at baseline. The study was reviewed by the competent ethics committee (Ethikkommission Nordwest- und Zentralschweiz; ref approval no. 2018–00976) and all procedures were in line with the ethical principles of the Declaration of Helsinki. The intervention study was registered in the WHO trial register (trial number: ISRCTN10469580). Participants received information about the general goals of the study and provided informed written consent before study entry. Participation in the study was voluntary and withdrawal or discontinuation possible at any time.

### 2.3 Data assessment and measures

Screening of inpatients took place in the first week after admission to inpatient treatment, baseline data assessment after 2–3 weeks after admission in one of the four involved clinics. All assessment procedures for the here presented data were identical for the entire sample. Two distinct assessment methods were employed to evaluate PA over two consecutive weeks: initially, participants self-reported their activity for the past week via the SIMPAQ, followed by a week where their PA was monitored via accelerometer device. In the following, we describe the assessments socio-demographic background, depressive symptom severity, self-reported PA, accelerometer-based PA, perceived fitness, estimated VO_2_max, exercise-based self-efficacy and outcome expectancies, exercise intention, and intrinsic exercise motivation.

#### 2.3.1 Sociodemographic characteristics

Participants reported on their gender at birth (male; female), age (years), civil status (single; married/in a partnership), nationality, highest level of education (none; obligatory schooling; apprenticeship; occupational training; high school; technical college/university; other); current employment status (employed; unemployed; retired), duration of employment (years), living with children (yes; no), and current smoking status (yes; no). For the assessment of body weight, a digital weighing scale (BC-545, Tanita, USA) was used (to the nearest 0.1 kg, in light cloths and without shoes). For the assessment of body height, a stadiometer (to the nearest 0.5 cm, without shoes) was used. Body Mass Index (BMI) was calculated as: weight (kg)/height (m^2^).

#### 2.3.2 Self-reported depressive symptom severity

Depressive symptom severity was assessed with the 21-item Beck Depression Inventory-II (BDI-II) (42, 43). The Beck Depression Inventory-II (BDI-II) is a widely utilized 21-item tool (42) designed to measure unipolar depression symptoms, including affective, cognitive, behavioral, and somatic aspects (e.g., “I am so unhappy/sad that I can't stand it”). Each item is rated on a scale from 0 to 3, indicating progressively severe symptoms of depression. Scores on the BDI-II can vary from 0 to 63, where a higher score reflects more severe depressive symptoms. The reliability and validity of this instrument have been well-documented in prior research (45). This study involved the comparison of three patient groups categorized by depression severity into mild (BDI-II scores of 0–19), moderate (BDI-II scores of 20–28), and severe (BDI-II scores of 29–63) depression, in line with established categories of depression severity (46).

#### 2.3.3 Self-reported physical activity

To assess self-reported PA, we employed the five-item Simple Physical Activity Questionnaire (SIMPAQ), a relatively new tool tailor-made for psychiatric patients (47). The SIMPAQ evaluated the average daily time spent in various physical activities over the preceding 7 days, referring to all domains of activity, including leisure, work, domestic, and transportation-related activities. Five items asked for participants' time (in hours) spent in bed, sitting, walking, participating in sports, and engaging in other activities, cumulatively approximating 24 h per day. Time spent walking and time spent exercising were combined to estimate total MVPA (23). To facilitate a more accurate comparison with the accelerometer data, we converted the daily time spent in various activities into minutes per week.

#### 2.3.4 Accelerometer-based physical activity

Accelerometer-based PA was assessed via an Actigraph wGT3x-BT device (Shalimar, FL, USA), positioned around the hip. Participants wore the device for seven successive days to capture an entire week. The sampling frequency was 60 Hertz (48). The raw data was processed with the ActiLife software V.6.13.4 (ActiLife, Shalimar, FL, USA). We used cut-off based analysis with defined cut-points in counts per minute (cpm) for MVPA (49).

For the cut-off based evaluation, we used an epoch length of 60 s epochs (50). We applied the following cut-points to assess the time spent in different activity categories: sedentary activity was defined as less than 610 counts per minute (cpm), light PA ranged from 611 to 2,689 cpm, moderate PA spanned from 2,690 to 6,166 cpm, vigorous PA from 6,167 to 9,642 cpm, and very vigorous PA was categorized as exceeding 9,643 cpm (49, 51). Whenever participants could not wear the monitor, like during swimming, they recorded the activity in a separate non-wear time sheet. Activities mentioned were classified as MVPA based on criteria from the PA compendium (52, 53). For the data to be considered for analysis, participants had to record a minimum of four days, comprising at least three weekdays and one weekend day (54). As per Herrmann et al. (55), only recordings with 8 h or more were deemed valid. The accuracy of the Actigraph devices has been documented in prior research (56).

#### 2.3.5 Perceived fitness

Participants rated their perceived fitness using a singular item (57). The question was: “How would you describe your physical condition?” They could select a score ranging from 1 (very poor) to 10 (excellent). This item's validity as a fitness perception indicator was backed by its high correlation with the 12-item Perceived Physical Fitness scale (58). It also showcased associations with objective fitness markers, well-being perceptions, and sleep patterns (57, 58).

#### 2.3.6 Cardiorespiratory fitness

Cardiorespiratory fitness (CRF) was estimated with the Åstrand indirect test of maximal oxygen uptake (59). The test was performed on a bicycle ergometer (Technogym, Bike Forma) at the same time of the day (starting between 8 and 10 am). The pedalling frequency was set at 50 revolutions per minute (rpm), while the workload was adjusted so that the heart rate was kept between 130 and 160 beats per minute (bpm) in participants younger than 40 years old and between 120 and 150 bpm in participants older than 40 years old. The Borg Rating of Perceived Exertion scale (60) was used to ensure that participants maintain their exercise intensity level at 13 or 14 (slightly strenuous). Following stabilization of heart rate after 5 or 6 min, maximal oxygen uptake (L/min) was estimated based on mean steady-state, sex and power-output, using a nomogram (59) and including a correction factor for age. Maximal oxygen uptake was expressed as VO_2_max (ml/kg/min), after correction of body weight. Previous studies have demonstrated the reliability and validity of the Åstrand nomogram and the linear extrapolation for deriving VO_2_max (61). We applied the following formula instead of the nomogram: for men: VO_2_max = [(0.00212 × workload × 6.12 + 0.299)/(0.769 × heart rate−48.5) × 100] × 1,000/weight/age correction factor; for women: VO_2_max = [(0.00193 × workload × 6.12 + 0.326)/(0.769 × heart rate−56.1) × 100] × 1,000/weight/age correction factor (62). This formula is based on sex, workload, and mean steady-state value of heart rate and is corrected for weight and age. Maximal oxygen uptake is expressed relative to body mass (ml/min/kg). Participants using beta-blockers at the time of the assessments (inpatients: *n* = 13, non-depressed controls: *n* = 2) were excluded from the analysis (63).

#### 2.3.7 Exercise-based self-efficacy

A trio of items touched upon starting, sustaining, and resuming exercise post-relapse, aiming to gauge self-efficacy in relation to exercise (e.g., “I feel confident to start with a new exercise activity”) (64). The answers ranged from 0 (not at all confident) to 5 (100% confident in myself). The three items were combined into a single (mean) score, with higher scores indicating greater self-efficacy. This scale proved to be psychometrically sound in previous studies (64) and was incorporated in prior intervention research (65–67).

#### 2.3.8 Exercise-based outcome expectancies

To assess exercise outcome expectancies, we used nine positively phrased (“pros”) and seven negatively framed items (“cons”) (68). These items, rated on a scale of 1 (not true) to 4 (completely true), were combined to form two distinct (mean) scores. This tool proved to have sound psychometric properties in previous research (68).

#### 2.3.9 Exercise intention

Exercise intention in the forthcoming weeks was assessed with the item “Strength of my intention to exercise regularly during the next few weeks and months”, with response options ranging from 0 (no intention) to 5 (very strong intention) (65, 69), and higher scores reflecting a stronger intention.

#### 2.3.10 Intrinsic exercise motivation

Intrinsic exercise motivation was assessed with the referring subscale of the self-concordance scale (SSK-scale) (69). The scale aligns with Sheldon and Elliot's self-concordance model (70). Three items were used to assess intrinsic motivation. Answered were provided on a scale from 1 (not at all true) to 6 (completely true).

### 2.4 Statistical analyses

SIMPAQ data were checked for univariate outliers, defined at >3 standard deviations below/above the mean (71). Univariate outliers were excluded from all further analyses. Kolmogorov-Smirnov tests were used to test normality of the data. In the results section, we first present descriptive statistics to describe the characteristics of the sample and the level of self-reported and accelerometer-based PA. Given the non-normal distribution of some parameters, Spearman correlation analyses were used to examine pairwise associations between self-reported and accelerometer-based PA (controlling for accelerometer wear-time). Spearman correlations were also used to examine bivariate relationships between the SIMPAQ subscales, perceived fitness, estimated VO_2_max, exercise-based self-efficacy and outcome expectancies, exercise intention, and intrinsic exercise motivation. All correlation analyses were performed separately in inpatients treated for MDD and non-depressed controls, and within each sample, separate analyses were run for women and men. Following the recommendations of Cohen (72), correlations of *rho* <0.30 were considered small, with *rho* = 0.30–0.50 as medium and *rho* >0.50 as large. All analyses were performed with SPSS Statistics 23 for Windows (IBM Corporation, Armonk NY, USA) and the level of significance was set at *p* < 0.05 across all analyses.

## 3 Results

### 3.1 Sample characteristics of inpatients treated for MDD and non-depressed controls

41 participants (16 inpatients treated for MDD and 25 non-depressed controls) were identified as univariate outliers (>3 standard deviations below/above the mean of each SIMPAQ category). Furthermore, 43 participants did not have valid accelerometer data. Among the non-depressed controls, 17 had excessively high depression scores. Additionally, 5 participants (across both groups) did not provide valid BDI scores. After these exclusions, the sample was comprised of 178 inpatients treated for MDD and 97 non-depressed controls. Table 1 provides an overview of the sample characteristics for the total sample, inpatients and non-depressed controls. Both groups had a similar distribution of gender: 45.5% inpatients treated for MDD were female, compared to 36.1% in the non-depressed control group. The average age was 39.04 years (SD = 13.31), with inpatients being significantly older than non-depressed controls (mean age 41.11 years, SD = 12.84, compared to 35.24 years, SD = 13.40). The majority spoke German as their first language (85.8%), identified as Swiss nationals (77.8%), and were single (69.8%). Within the inpatient group, 34.3% had a first-episode MDD diagnosis (F32), 64.0% had recurrent MDD (F33 diagnosis), and 1.7% were diagnosed with bipolar disorder type II (F31.80). Notably, 162 inpatients were on antidepressants (no controls were on antidepressant medication). All participants met the screening criteria for self-reported insufficient PA; however, at baseline, 162 participants accumulated 150 min or more of accelerometer-based MVPA per week.

**Table 1:** Sample characteristics.

		Total sample (*N *= 275)		Inpatients treated for MDD (*n *= 178)		Non-depressed controls (*n *= 97)		* *	
		*n*	%	*n*	%	*n*	%	Chi^2^	ϕ
Sex (female)		116	42.2	81	45.5	35	36.1	2.29	.09
Language (German as first language)		236	85.8	156	87.6	80	82.5	1.38	.07
Nationality (Swiss)		214	77.8	149	83.7	65	67.0	10.14***	.19***
Marital status (single)		192	69.8	125	65.1	67	69.1	0.04	.01
Level of education (higher education)		132	48.0	63	35.4	69	71.1	32.13***	.34***
Children living at home (yes)		63	22.9	45	25.3	18	18.6	1.61	-.08
Antidepressant intake (yes)		162	58.9	162	91.0	0	0.0	214.84***	-.88***
Smoking (yes)		91	33.1	72	40.4	19	19.6	12.34***	-.21***
Depression subtype	Single episode (F32)	–	–	61	34.3	–	–	–	–
	Recurrent MDD (F33)	–	–	114	64.0	–	–	–	–
	Bipolar disorder type II (F31.8)	–	–	3	1.7	–	–	–	–
		*M*	*SD*	*M*	*SD*	*M*	*SD*	*F*	Eta^2^
Age (in years)		39.04	13.31	41.11	12.84	35.24	13.40	12.73***	.05
Height (in cm)		170.63	9.37	171.13	9.39	169.73	9.32	1.40	.01
Weight (in kg)		76.60	19.36	80.84	19.71	68.81	16.09	26.52***	.09
Body mass index (kg/m^2^)		26.23	5.93	27.50	6.03	23.87	4.97	25.44***	.09
Employment rate (in hours/week)		23.64	18.70	23.68	18.90	23.57	18.42	0.00	.00
Years of job experience (in years)		16.51	13.14	18.66	12.56	12.57	13.32	14.15***	.05
Depressive symptom severity (at baseline)		–	–	28.42	9.36	–	–	–	–
Duration of current episode (in weeks)^a^		–	–	34.30	36.73	–	–	–	–
Number of prior depressive episodes^b^		–	–	3.14	6.32	–	–	–	–
Age of onset of depression (in years)^c^		–	–	29.18	14.09	–	–	–	–

MDD, major depressive disorders.

^a^ 32 values missing.

^b^ 17 values missing.

^c^ 16 values missing.

*** *p* < .001.

### 3.2 Descriptive statistics for inpatients treated for MDD and non-depressed controls

Table 2 presents the descriptive statistics for self-reported PA, accelerometer-based PA, self-perceived fitness, and estimated VO_2_max, exercise-related self-efficacy and outcome expectancies, exercise intention, intrinsic exercise motivation, separately for inpatients treated for MDD, non-depressed controls. In view of the fact that the two groups were assessed in different life circumstances (inpatients: during hospitalisation with structured therapy; controls: in their natural environment), we deliberately refrained from using inferential statistics to calculate differences.

**Table 2:** Descriptive statistics for inpatients treated for MDD and non-depressed controls in main study variables.

	Total sample (*n *= 275)		Inpatients treated for MDD (*n *= 178)		Non-depressed controls (*n *= 97)	
	*M*	*SD*	*M*	*SD*	*M*	*SD*
SIMPAQ						
Time in bed (min/day)	502.8	69.0	513.6	67.2	482.4	69.0
Sedentary time (min/day)	663.6	157.8	665.4	144.0	660.0	181.8
Time spent walking (min/day)	163.2	119.4	153.6	100.2	181.8	148.2
Exercise and sport (min/day)	18.0	19.8	20.4	19.8	13.2	19.2
All other physical activities (min/day)	21.0	33.0	12.0	25.2	37.2	39.0
Accelerometry						
Sedentary time (min/day)	574.4	92.0	577.4	90.9	568.7	94.4
Light physical activity (min/day)	186.7	58.3	173.8	47.2	211.1	68.9
MVPA (min/day)	55.7	27.8	56.9	27.4	53.2	28.5
Steps (per day)	7,902.9	2,847.2	7,997.9	2,797.9	7,722.5	2,946.3
Exercise determinants						
Self-efficacy	3.59	0.97	3.48	0.99	3.79	0.90
Positive outcome expectancies	3.11	0.46	3.20	0.42	2.94	0.49
Negative outcome expectancies	1.96	0.51	1.98	0.51	1.91	0.50
Exercise intention	3.53	1.17	3.72	1.06	3.16	1.28
Intrinsic motivation	3.67	1.08	3.73	1.09	3.57	1.06
Fitness						
Self-perceived fitness	4.05	1.73	3.65	1.59	4.81	1.73
Estimated VO_2_max	35.36	9.64	33.18	9.50	39.46	8.55

MDD, major depressive disorders; SIMPAQ, Simple Physical Activity Questionnaire; MVPA, moderate-to-vigorous physical activity; VO_2_max, maximal oxygen uptake.

### 3.3 Correspondence of SIMPAQ with accelerometer-based physical activity

Table 3 displays the bivariate correlations between SIMPAQ subscores and accelerometer-based PA for the total sample, inpatients treated for MDD and non-depressed controls. Broadly speaking, correlations between the SIMPAQ subscores and accelerometer-based PA were ranging from non-existent (zero) to moderate, with correlations only marginally differing between inpatients treated for MDD and non-depressed controls.

**Table 3:** Spearman correlations between SIMPAQ measures and accelerometer data for the total sample, and separately for inpatients treated for MDD and non-depressed controls.

Total sample (*N* = 275)	Sedentary time	LPA	MVPA	Steps
SIMPAQ	*rho*	*rho*	*rho*	*rho*
Time in bed	−.09	−.24***	−.10	−.13*
Sedentary time	.19***	−.14*	−.05	−.09
Time spent walking	−.12	.25***	.07	.15*
Exercise and sport	.03	−.04	.09	.06
Other physical activities	−.07	.15*	.07	.01
MVPA	−.11	.25***	.10	.18*
Inpatients treated for MDD (*n* = 178)				
SIMPAQ	*rho*	*rho*	*rho*	*rho*
Time in bed	−.03	−.22**	−.15*	−.16*
Sedentary time	.10	−.03	−.05	−.01
Time spent walking	−.05	.13	.10	.10
Exercise and sport	−.03	.08	.14	.13
Other physical activities	−.04	.09	.11	.05
MVPA	−.06	.14	.15*	.15*
Non-depressed controls (*n* = 97)				
SIMPAQ	*rho*	*rho*	*rho*	*rho*
Time in bed	−.24*	−.14	−.03	−.10
Sedentary time	.31**	−.37***	−.08	−.23*
Time spent walking	−.19	.46***	.03	.24*
Exercise and sport	.09	−.07	.01	−.08
Other physical activities	−.09	.00	.02	−.06
MVPA	−.17	.46***	.03	.25*

MDD, major depressive disorders; SIMPAQ, Simple Physical Activity Questionnaire; MVPA, moderate-to-vigorous physical activity.

* *p* < .05.

** *p* < .01.

*** *p* < .001.

For the total sample, correlations were small and mostly non-significant. There was a small significant positive correlation between sedentary time score from both measures. Similarly, time spent walking, as assessed with the SIMPAQ, correlated positively with accelerometer-based light PA. Accelerometer-based MVPA was not associated with any of the SIMPAQ subscores. Finally, two small positive correlations were found for accelerometer-based steps and the two SIMPAQ subscores “walking” and “MVPA”.

For inpatients, SIMPAQ-based time spent in bed correlated negatively with accelerometer-based LPA, MVPA and steps. Furthermore, SIMPAQ-based MVPA correlated positively (but weakly) with accelerometer-based MVPA and steps. All other correlations were not statistically significant.

For the non-depressed controls, two positive correlations were found between SIMPAQ-based subscores “walking” and “MVPA” and accelerometer-based light PA. Against our expectations, accelerometer-based MVPA was not associated with any of the SIMPAQ subscores. Finally, accelerometer-based steps correlated positively (but weakly) with the SIMPAQ-based “walking” and “MVPA”.

In summary, few statistically significant and only weak-to-moderate correlations were found between self-reported (SIMPAQ-based) and accelerometer-based PA indicators. Especially, only a low level of correspondence was observed between the SIMPAQ and accelerometer-based MVPA.

### 3.4 Correlations of SIMPAQ subscores with fitness measures

Table 4 presents the bivariate correlations between SIMPAQ subscores and self-perceived fitness, and estimated VO_2_max, for the total sample and separately for inpatients treated for MDD and non-depressed controls. In the total sample, a weak positive correlation was found between SIMPAQ-based MVPA and perceived fitness, whereas in both subpopulations, a weak positive correlation occurred between SIMPAQ-based exercise/sport and perceived fitness. Estimated VO_2_max was not correlated with any of the SIMPAQ subscores.

**Table 4:** Spearman correlations between SIMPAQ measures, fitness measures, and exercise determinants for the total sample, inpatients treated for MDD and non-depressed controls.

Total sample (*N* = 275)	SIMPAQ					
	Time in bed	Sedentary time	Walking	Exercise/sport	Other activities	MVPA
	*rho*	*rho*	*rho*	*rho*	*rho*	*rho*
Fitness						
Self-perceived fitness	−.09	−.05	.09	.09	.08	.12*
Estimated VO_2_max	−.05	.06	.05	.07	−.01	.08
Exercise determinants						
Self-efficacy	−.04	.03	.04	.06	.02	.05
Positive outcome expectancies	.10	−.02	−.03	.09	−.10	−.01
Negative outcome expectancies	.03	.02	−.04	−.14*	.03	−.07
Exercise intention	.08	−.05	.04	.24***	−.15*	.08
Intrinsic exercise motivation	.01	−.06	.13*	.19**	−.02	.18**
Inpatients treated for MDD (*n* = 178)						
	*rho*	*rho*	*rho*	*rho*	*rho*	*rho*
Fitness						
Self-perceived fitness	−.03	−.02	.11	.16*	−.07	.14
Estimated VO_2_max	.05	−.01	.11	.12	−.13	.14
Exercise determinants						
Self-efficacy	−.05	.02	.13	.04	−.09	.12
Positive outcome expectancies	.03	.01	−.06	−.04	.08	−.05
Negative outcome expectancies	−.01	.07	−.07	−.17*	.18*	−.10
Exercise intention	.01	−.08	.10	.21**	−.07	.13
Intrinsic exercise motivation	−.01	−.03	.18*	.14	−.05	.22**
Non-depressed controls (*n* = 97)						
	*rho*	*rho*	*rho*	*rho*	*rho*	*rho*
Fitness						
Self-perceived fitness	−.01	−.11	.07	.21*	.09	.11
Estimated VO_2_max	−.09	.15	−.04	.17	.04	−.01
Exercise determinants						
Self-efficacy	.10	.03	−.12	.20	.06	−.09
Positive outcome expectancies	.11	−.05	.00	.11	−.10	.01
Negative outcome expectancies	.02	−.05	.01	−.17	−.11	−.02
Exercise intention	.10	.01	−.04	.16	−.13	−.01
Intrinsic exercise motivation	.02	−.11	.06	.28**	.08	.10

MDD, major depressive disorders; SIMPAQ, Simple Physical Activity Questionnaire; MVPA, moderate-to-vigorous physical activity; VO_2_max, maximal oxygen uptake.

* *p* < .05.

** *p* < .01.

*** *p* < .001.

### 3.5 Correlations of SIMPAQ subscores with exercise determinants

Table 4 presents the bivariate correlations between SIMPAQ subscores and exercise determinants. Overall, only few statistically significant correlations were found. In inpatients, SIMPAQ-based exercise/sport correlated negatively with negative outcome expectancies and positively with exercise intention. SIMPAQ-based MVPA was associated with intrinsic exercise motivation. However, all three correlations were weak. In non-depressed controls, a positive but weak correlation was found between SIMPAQ-based exercise/sport and intrinsic exercise motivation. All other correlations were not statistically significant.

## 4 Discussion

This study aimed to address challenges in the assessment of PA. Therefore, we examined the correspondence between self-reported (SIMPAQ-based) and accelerometer-based PA in inpatients treated for MDD and non-depressed controls. We further examined the association between SIMPAQ-based PA, fitness measures and exercise determinants.

The key findings of the present study are that the subsequently assessed self-reported and accelerometer-based PA were only weakly associated with each other in both subpopulations. This result underscores the potential discrepancy between self-reported and device-based PA levels, especially within the MDD context (32, 73, 74). This finding is in line with previous research (33), highlighting the need for further research in accurate assessment tools for inpatients treated with MDD or non-depressed individuals with initially low PA levels.

Three hypotheses were formulated and each of them will now be addressed in turn. Hypothesis 1 expected weak-to-moderate relationships between self-reported and accelerometer-based PA in inpatients treated for MDD and non-depressed controls. Although some significant correlations occurred, our data did not support this hypothesis. Overall, there was a very poor correspondence between self-reported and accelerometer-based PA measures in both populations. Among inpatients, the reasons for these discrepancies with self-report measures might be attributable to cognitive biases associated with depression (32), including self-regulatory effects (75, 76). However, a similar pattern was observed in non-depressed controls, which is at odds with a previous study with a more physically active population (exercise and sport students), in which higher correlations were found between the SIMPAQ and the same accelerometer devices (36). It is thus possible that the SIMPAQ works better in people with higher PA levels; however, it is also possible that the higher level of agreement is due to the fact that in the study by Schilling et al. (36) the two assessments periods were fully in line, whereas in our study the two instruments were used one week apart. In the present study, this methodological issue probably had a stronger influence on the control group, as PA behavior may fluctuate more in a natural environment. The effect should be less significant in inpatients, as their everyday life is more structured due to the therapy programmes and often includes structured sport and exercise activities (77, 78).

Only limited support was found for our second hypothesis, which expected positive associations between self-reported PA and perceived fitness as well as estimated VO_2_max. Thus, few statistically significant associations were found between SIMPAQ subscores and perceived fitness, whereas SIMPAQ subscores and estimated VO_2_max were fully unrelated. Studies have shown that objective fitness measures, such as VO_2_max, often show low correlations with subjective fitness parameters (79). This discrepancy is particularly evident in populations with depression, where negative cognitive biases can distort self-assessments of fitness despite regular PA. Research suggests that cognitive factors like processing speed and working memory significantly influence how individuals perceive their health and fitness, highlighting the importance of considering cognitive processes in the evaluation and improvement of self-perceived fitness (80). Hypothesis 3 anticipated that some SIMPAQ measures (especially the “exercise/sport” subscore) would be associated with cognitive determinants of exercise behavior, such as exercise-related self-efficacy, outcome expectancies, exercise intention, and intrinsic motivation towards exercise. While some expected relationships emerged, others did not. Moreover, the strength of the associations was weak at best. Notably, self-efficacy and positive outcome expectancies were not associated with SIMPAQ-based PA and exercise behaviour across both groups. The missing relationship between self-efficacy and PA behavior in inpatients treated for MDD underscores the potential need for future research to differentiate subtypes of self-efficacy (task, maintenance and recovery self-efficacy) (81). Regarding outcome expectancies, there is no consensus in the literature. While some studies indicate that depressed individuals have lower positive outcome expectancies compared to non-depressed individuals (82), others suggest they actually exhibit reduced negative outcome expectancies, instead (81). Descriptively, inpatients treated for MDD reported higher levels of positive outcome expectancies compared to non-depressed controls in the present study. However, between-group differences must be interpreted with utmost caution as they may be attributable to a selection bias (in our study, all inpatients were willing to take part in a PA counselling intervention). As expected, in inpatients treated for MDD, stronger exercise intentions correlated positively with self-reported exercise and sport. By contrast, it is intriguing to note that their intrinsic motivation for exercise was not significantly correlated with their exercise and sport behavior (which was the case in non-depressed controls). This observation might be due to the fact that inpatients were assessed in a clinical setting, in which some exercise and sport activities are prescribed by a physician. As a result, participation in these activities might be based more on extrinsic than intrinsic motivation.

Several potential limitations of the current study are worth noting. Firstly, the findings may not be generalizable to the broader population of patients with MDD (e.g., outpatients). Secondly, as mentioned above, the measurement periods for the two instruments, SIMPAQ and accelerometers, were not fully synchronised. Thus, accelerometer-based data were prospectively gathered, whereas the SIMPAQ focused on PA patterns during the last week, as they were carried out on two consecutive weeks. This might have reduced the level of correspondence between the two measures, and explain why correlations were smaller than in previous validation studies. However, in research and clinical practice, both measures are used to assess habitual PA behavior. Thus, some level of correspondence would be expected even if the measures are not fully synchronized, particularly in inpatients, where daily routines are structured and subject to fewer changes than in normal life. Thirdly, the cross-sectional nature of our data does not enable us to examine causality between exercise determinants and actual behaviour. Lastly, given that inpatients and controls were assessed in different settings and due to a possible selection bias, we decided against calculating group differences based on inferential statistics. On the other hand, all inpatients were diagnosed with depression via structured clinical interviews by a psychiatrist, ensuring a rigorous and standardized assessment of pathology. Although structured clinical interviews are the only well-validated method to establish a clinical diagnosis of depression, in many studies, researchers used self-report questionnaires to assess depressive symptoms (83).

## 5 Conclusion

The present study shows a low level of correspondence between self-reported and accelerometer-based PA, applied on two different, subsequent weeks, in both inpatients treated for MDD, and non-depressed controls. Our data highlight that the assessment of PA remains a challenge in psychiatric patients and individuals with low PA levels. While the SIMPAQ was initially designed to overcome the shortcomings of existing PA questionnaires (particularly the IPAQ), our data points towards the ongoing need to refine and improve PA questionnaires like the SIMPAQ for a more accurate PA assessment in psychiatric populations. Additionally, the discrepancy between self-reported and accelerometer-based PA underlines the necessity for a diversified assessment of PA. The availability of accurate PA questionnaires is important from a clinical perspective, as the development of a physically active lifestyle has become an important treatment goal in psychiatry (84).

## Data Availability

The datasets used and/or analyzed during the current study are available from the corresponding author on reasonable request via the Ethics Committee of Northwestern and Central Switzerland (EKNZ). At the time of obtaining ethical clearance for the present study from the EKNZ, and in line with Swiss laws, we stated that only authorized researchers who are directly involved in the present project will have access to the raw data. Accordingly, and in line with this statement, we cannot grant access to the data for third parties, unless this is officially approved by the EKNZ.
